# Manual of Chemistry

**Published:** 1845-01

**Authors:** 


*A Manual of Chemistry, &c.* By Lewis C. Beck, M. D., Professor of Chemistry. 12mo. New York, 1844.

We have before us the fourth edition of this manual, which is presented to the public in a new and much improved form, having undergone a complete revision, since the publication of the third edition, by which it has been rendered more in accordance with the present state of chemical science. As a *Manual* of chemistry, it offers no claims to be considered as a complete and detailed view of the whole science of chemistry, but, by embodying in a condensed form the more important facts and views in this branch of knowledge, aims at opening the way for additional instruction—and serving for an introduction to more copious and more extended works. In these views the author has succeeded to a considerable extent, by giving in a terse and comprehensive style, descriptions of the various processes, notices of general qualities and explanations of the most important re-actions, which are connected with the study both of elementary and compound bodies. In most instances the prominent characters, and the points demanding particular attention, are placed in separate paragraphs, each with an appropriate head—thus giving to them a bold and distinct relief, and facilitating the means of reference, by affording prominent points to a hasty glance, without any necessity for a perusal of the paragraph itself.

We notice with pleasure that the author has adopted whole numbers for the combining weights, which even should they not "eventually be found accurately to express the atomic weights," yet they afford greater facilities in teaching, and do not tend to load the mind of the student in an unnecessary manner. In fact, in learning the science, it is more important to obtain a strict comprehension of fundamental principles, than to acquire a mathematical accuracy of detail.

The introduction of symbols is another improvement, which could not have been omitted without injury to the general character of the work.

Numerous wood cuts exhibiting the more simple forms of apparatus, in which the various processes may be carried on, are also added, and will afford facilities to those who are desirous of practical knowledge, by pointing out cheap and easy modes, by which it may be obtained. We may also notice as tending to the same end, the constant reference made, in connection with each subject, to more extended and elaborate treatises; not only indicating the origin and authority of the facts stated, but also enabling the inquirer to search out in detail all the information of which only an outline could be given in the text.

We perceive a few inaccuracies which the author has overlooked, but which are of minor importance, and which, even with the greatest care, would seem inevitable in a work extending over so much ground as chemical science now occupies.

